# Supplementary material for: De novo design of immunoglobulin-like domains
Source: Nat Commun. 2022 Oct 3;13:5661. doi: 10.1038/s41467-022-33004-6 (PMC9530121; doi:10.1038/s41467-022-33004-6)
Supplement: Supplementary file 1 — Supplementary Information [file 41467_2022_33004_MOESM1_ESM.pdf]

Supplementary Information

De Novo Design of Immunoglobulin-like Domains

Tamuka M. Chidyausiku[1,2,7]†‡, Soraia R. Mendes[3]†, Jason C. Klima[1,2]§, Marta Nadal[4], Ulrich Eckhard[3], Jorge Roel-Touris[4], Scott Houliston[5,6], Tibisay Guevara[3], Hugh K. Haddox[2], Adam Moyer[2], Cheryl H. Arrowsmith[5,6], F. Xavier Gomis-Rüth[3]*, David Baker[1,2,7]*, Enrique Marcos[4]*

[1]Department of Biochemistry, University of Washington, Seattle, WA 98195, USA
[2]Institute for Protein Design, University of Washington, Seattle, WA 98195, USA
[3]Proteolysis Laboratory, Department of Structural Biology, Molecular Biology Institute of Barcelona (IBMB-CSIC), Baldiri Reixac 15, 08028 Barcelona, Spain
[4]Protein Design and Modeling Lab, Department of Structural Biology, Molecular Biology Institute of Barcelona (IBMB-CSIC), Baldiri Reixac 15, 08028 Barcelona, Spain
[5]Structural Genomics Consortium, University of Toronto, Toronto, ON, M5G 1L7, Canada
[6]Princess Margaret Cancer Centre and Department of Medical Biophysics, University of Toronto, Toronto, ON, M5G 2M9, Canada
[7]Howard Hughes Medical Institute, University of Washington, Seattle, WA 98195, USA

† These authors contributed equally to this work
‡ Present address: Novartis Institutes for BioMedical Research Inc., San Diego, CA 92121, USA
§ Present address: Encodia, Inc., San Diego, CA 92121, USA
*Corresponding authors: embcri@ibmb.csic.es, dabaker@uw.edu, xgrcri@ibmb.csic.es

a ABEGO definition

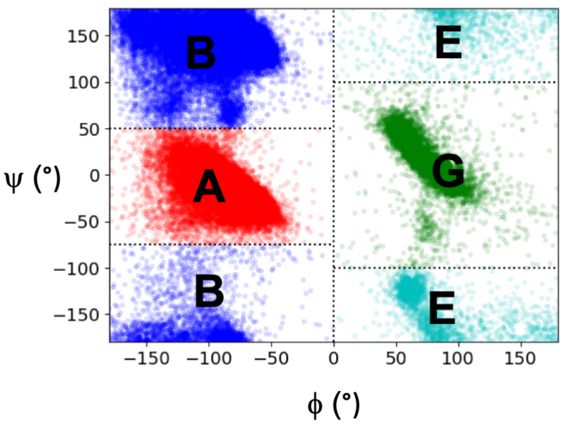

b β-arch sidechain orientations

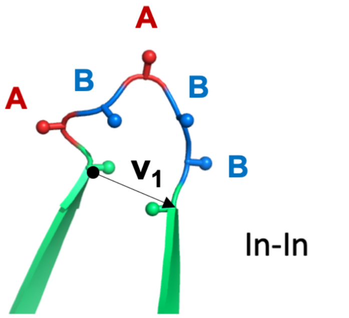

c

In-In

ABEGO	Frequency
ABB	118
BBG	81
ABABB	79
BBGBB	54
ABBG	24

In-Out

ABEGO	Frequency
BBGB	216
BAAB	29
ABA	25
ABBBA	24
BAABA	18

Out-Out

ABEGO	Frequency
BGB	68
AAB	34
BBBGB	33
AAABA	25
BABB	22

Out-In

ABEGO	Frequency
BABB	44
BGBB	34
AAA	22
GBB	21
BBAA	14

Supplementary Fig. 1. Frequently observed β-arch loops in naturally occurring protein structures. **a,** The Ramachandran plot is conveniently discretized in ABEGO torsion bins describing local backbone geometry at the residue level ("A", right-handed α-helix region (red); "B", extended region (blue); "E", extended region with positive ϕ (cyan); "G", left-handed α-helix region (green); and "O", if the peptide bond dihedral angle (ω) deviates from planarity). **b**, Definition of the β-arch sidechain orientation based on the relative orientation between the translation vector ($\mathbf{v}_1$) and the $\mathbf{C}_\alpha$-$\mathbf{C}_\beta$ vector of the two adjacent β-strand residues. If the $\mathbf{C}_\alpha$-$\mathbf{C}_\beta$ vector of the preceding residue is oriented in the same direction as $\mathbf{v}_1$, then the sidechain orientation is considered to point inwards ("In"), otherwise it is considered to point outwards ("Out"). The same applies to the residue following the loop but considering $-\mathbf{v}_1$ as the translation vector. Loop positions are colored according to their ABEGO bin, as shown in (**a**). **c**, β-arch loops (ranging between 3 and 5 residues) spanning the four possible sidechain orientations that are most frequently observed in a non-redundant set of naturally occurring protein structures. Source data are provided as a Source Data file.

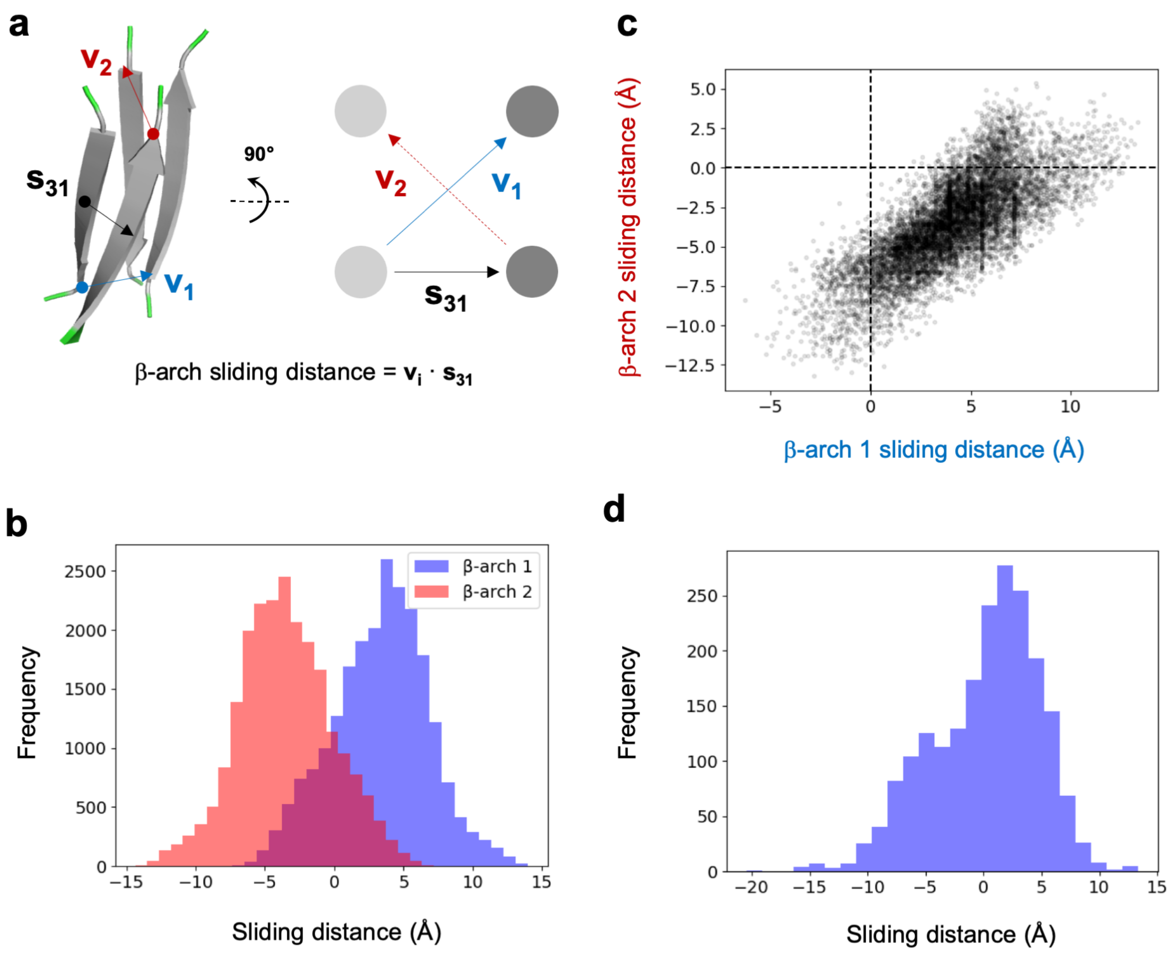

Supplementary Fig. 2. Coupling between the two β-arches forming cross-β motifs. a, β-arch sliding distance definition. Cartoon representation (left) and diagram (right) of a cross-β motif. We define $\mathbf{v_1}$ and $\mathbf{v_2}$ as the translation vectors connecting the C_α atoms of the residues preceding and following β-arch loops 1 and 2, respectively; and the $\mathbf{S_{31}}$ vector between the centers of the two N-terminal β-strands (1 and 3). The sliding distance is the projection of the β-arch translation vectors onto the $\mathbf{S_{31}}$ vector. **b,** Distribution of β-arch sliding distances in cross-β motifs generated by Rosetta folding simulations. In general, cross-β motifs tend to have positive and negative sliding distances for β-arches 1 and 2. **c**, Correlation between the two β-arch sliding distances in simulated cross-β motifs with low twist rotations (between -10° and 10°). **d**, Distribution of β-arch sliding distances in β-arch loops from naturally occurring protein structures. Source data are provided as a Source Data file.

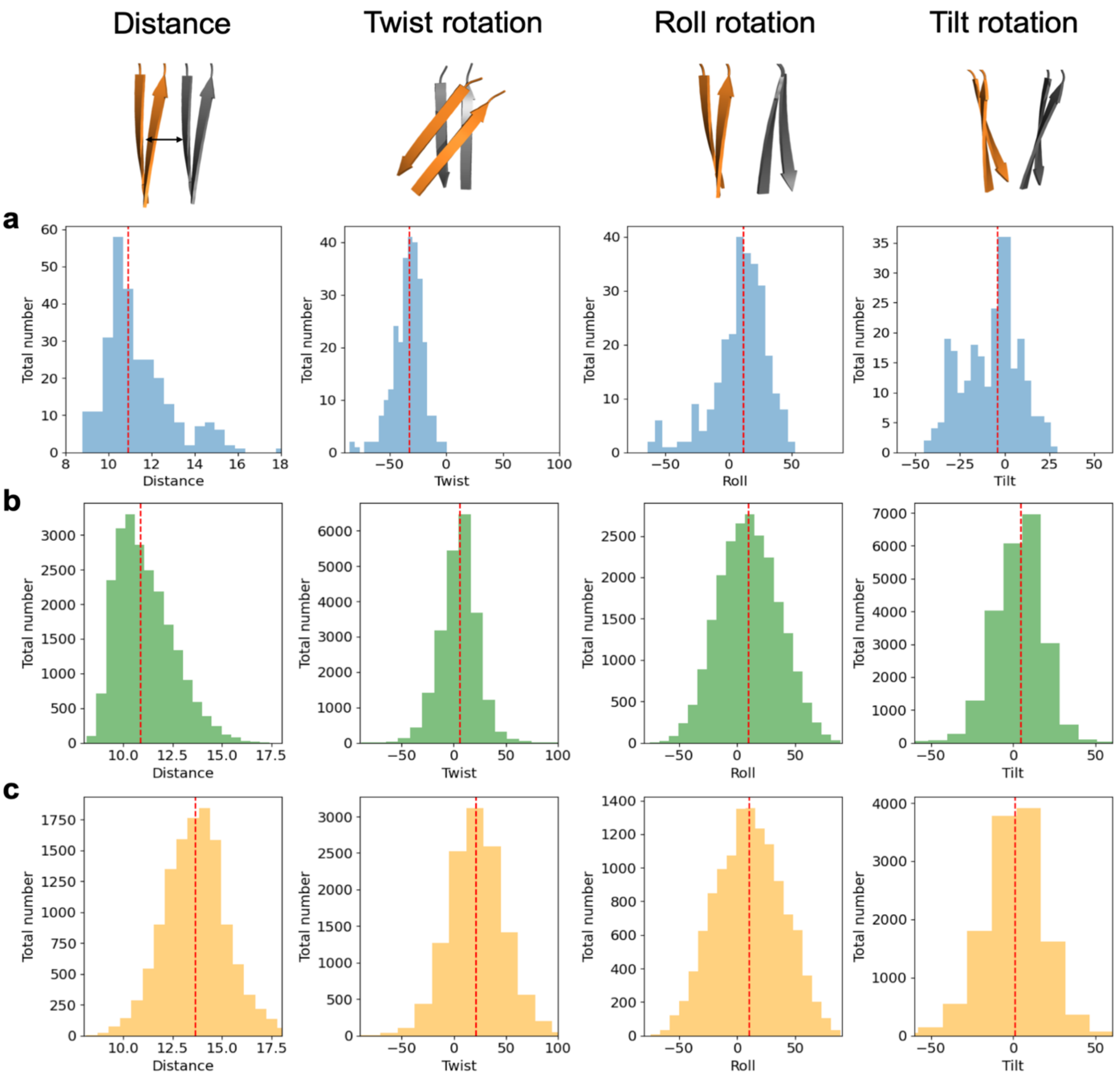

Supplementary Fig. 3. Distributions of cross-β geometrical parameters obtained from naturally occurring Ig domains and Rosetta folding simulations. a, Median (red dotted line) and median absolute deviations for each parameter: distance (10.9 ± 0.8 Å), twist (-32.1 ± 7.7°), roll (12.0 ± 12.2°) and tilt (-4.0 ± 11.1°). Distributions correspond to a set of 275 natural Ig domains with sequence identity below 40%. **b**, Median (red dotted line) and median absolute deviations for each parameter: distance (10.9 ± 1.0 Å), twist (5.7 ± 11.0°), roll (9.7 ± 18.0°) and tilt (4.5 ± 9.6°). Distributions correspond to 22,507 cross-β motif models generated by Rosetta folding simulations exploring different combinations of strand lengths (5-7 residues) and frequently observed β-arch loops (3-5 residues). **c**, Median (red dotted line) and median absolute deviations for each parameter: distance (13.7 ± 1.0 Å), twist (21.1 ± 17.2°), roll (10.3 ± 20.4°) and tilt (1.2 ± 11.2°). Distributions correspond to 12,335 cross-β motif models generated by Rosetta fragment assembly simulations exploring different combinations of strand lengths (5-7 residues) and β-arch helices (3-5 residues). Source data are provided as a Source Data file.

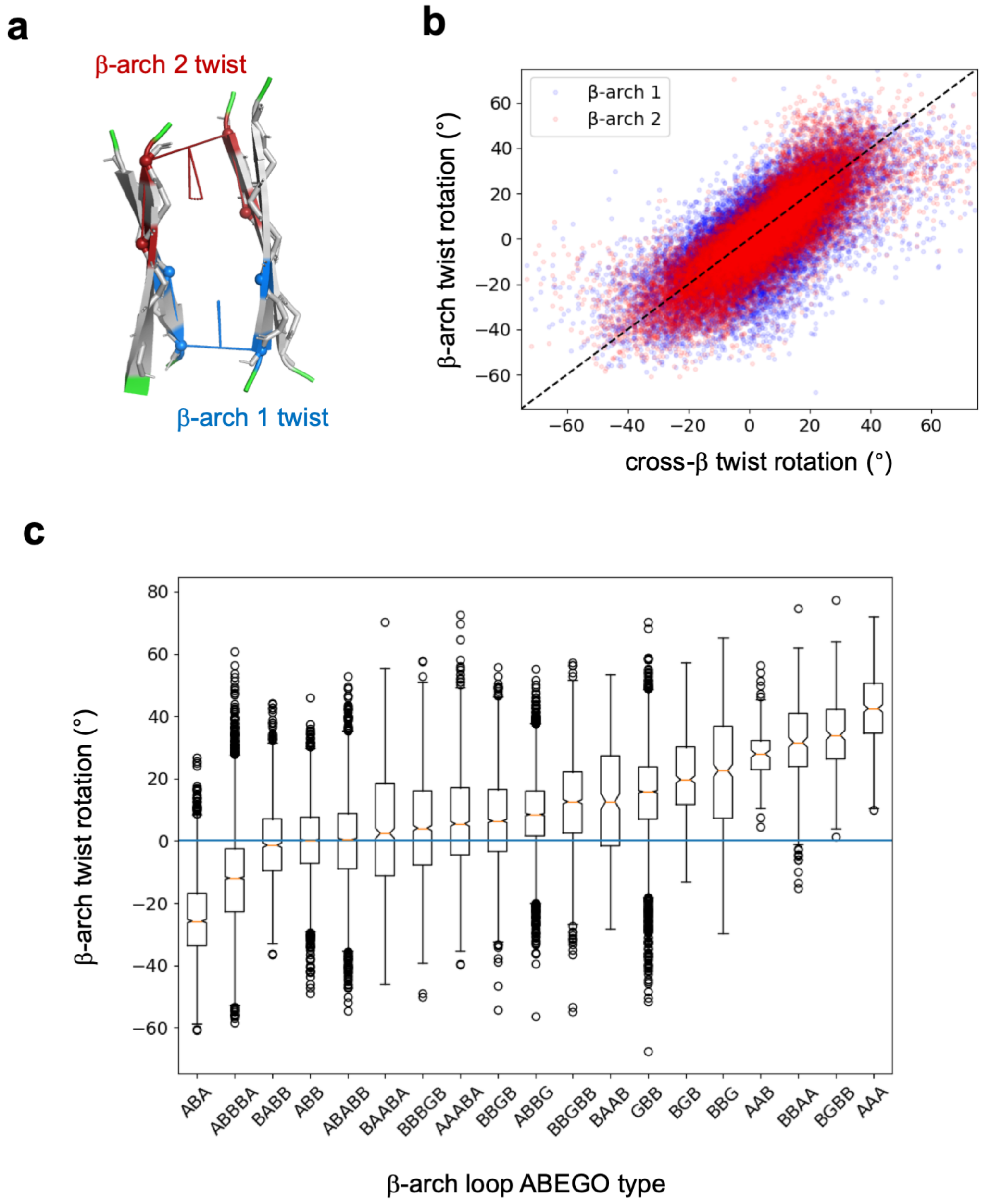

Supplementary Fig. 4. β-arch loops can twist cross-β motifs in different directions depending on their geometry. **a**, Definition of β-arch twist based on the dihedral angle formed between the α-carbons C_α (i-2), C_α (i), C_α (j) and C_α (j+2); where i and j correspond to the residues preceding and following the β-arch loop. **b**, Correlation between β-arch loop twisting and the cross-β twist rotation obtained from Rosetta folding simulations. **c**, Distributions of β-arch twist values for loops with frequently observed ABEGO torsion bins forming cross-β motifs in Rosetta folding simulations, sampling both positive and negative rotations. Boxplots show the 25% percentile, median, 75% percentile, whiskers extending 1.5 times the interquartile range and outliers outside the whiskers range. The number of samples of each ABEGO boxplot ranges between 167 and 6179, which also depends on the differential efficiencies in forming cross-β motifs as observed from simulations. Source data are provided as a Source Data file.

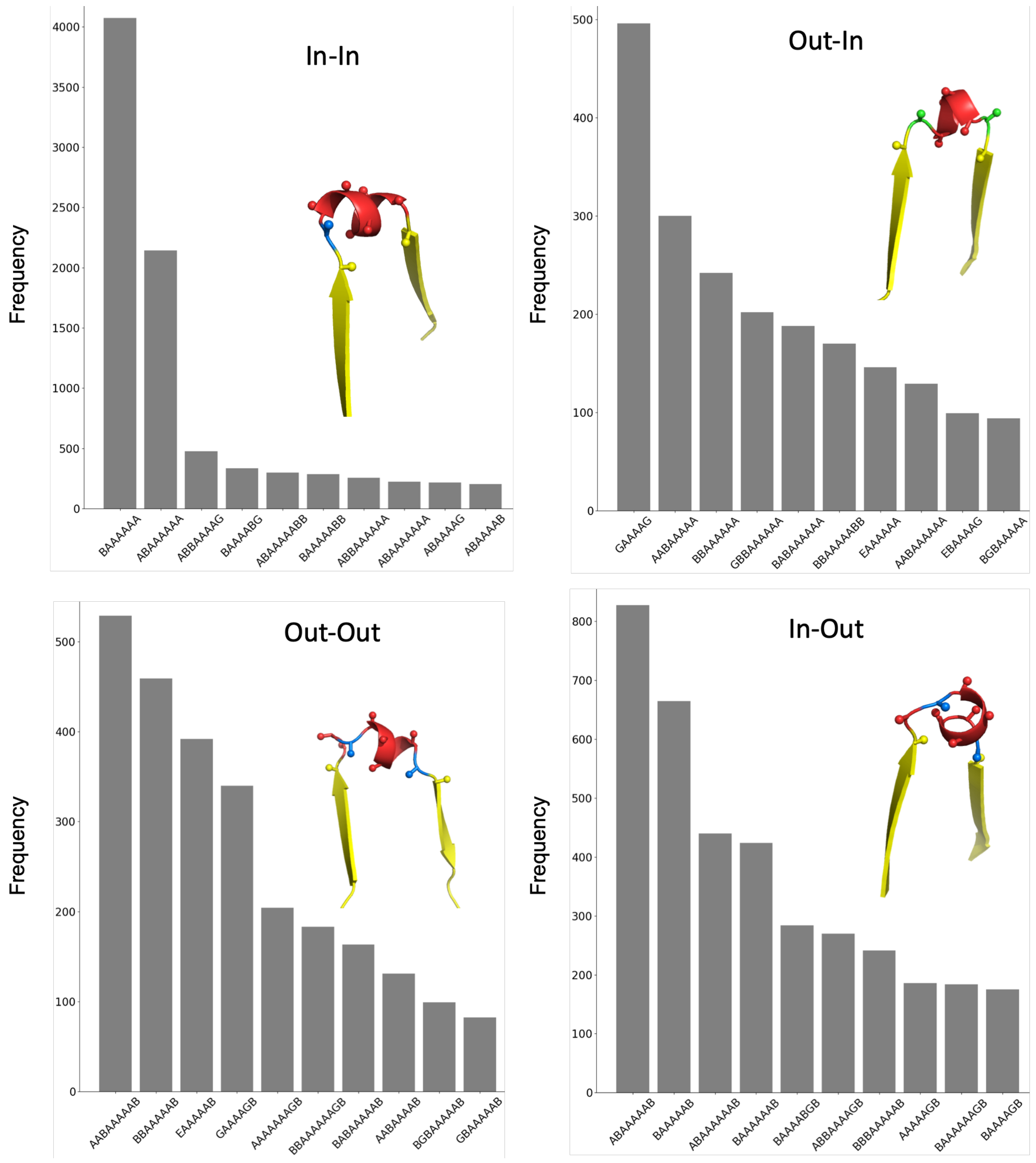

Supplementary Fig. 5. β-arch helices favoring cross-β motifs obtained from Rosetta folding simulations. The 10 most frequently observed loop-helix-loop ABEGO patterns of each possible sidechain orientation are shown on the horizontal axes. Frequencies are calculated as the total number of counts across β-arches from all generated cross-β motifs by Rosetta folding simulations with a sequence-independent model. Cross-β motif examples for the most frequently observed ABEGO pattern of each sidechain orientation is shown and color-coded as in Supplementary Fig. 1a, with the preceding and following β-strands in yellow. Most ABEGO patterns have a "B" torsion in the residue preceding the helix, which is typically observed at the start of α-helices as it provides N-terminal hydrogen bond capping. Source data are provided as a Source Data file.

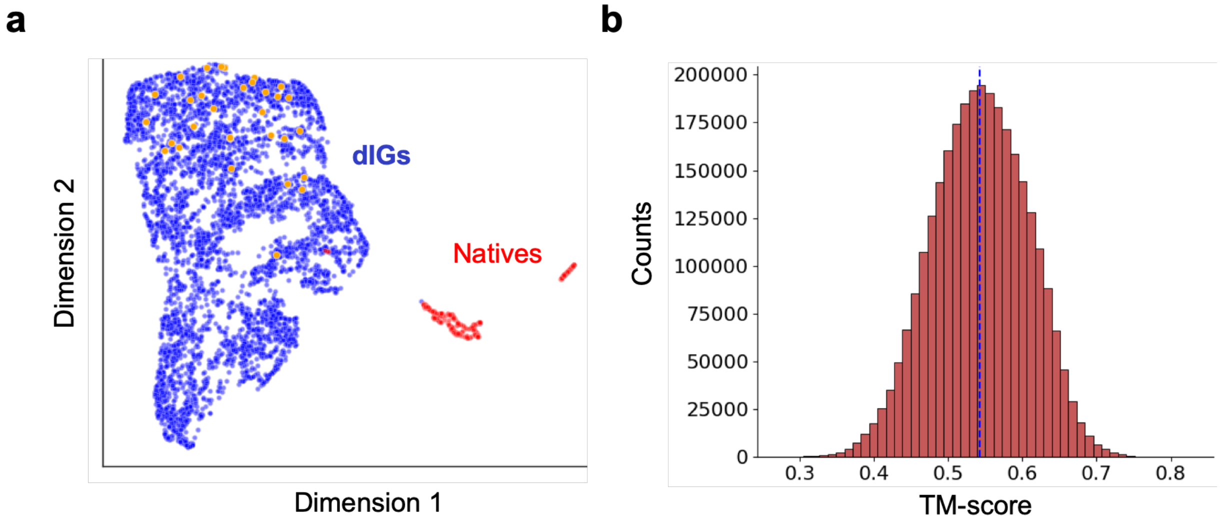

Supplementary Fig. 6. Structural diversity of the designed proteins and their comparison to natural Ig-like domains. a, Uniform manifold approximation and projection (UMAP) analysis of computationally designed (blue) and naturally occurring (red) Ig domains based on pairwise distances calculated as the TM-score (*1*). Experimentally tested designs are shown in orange. The designs broadly sample a structural space distinct from natural Ig proteins. **b**, Distribution of TM-scores between designs and natural Ig domains (mean 0.54 ± 0.06 s.d. (dashed blue line)).

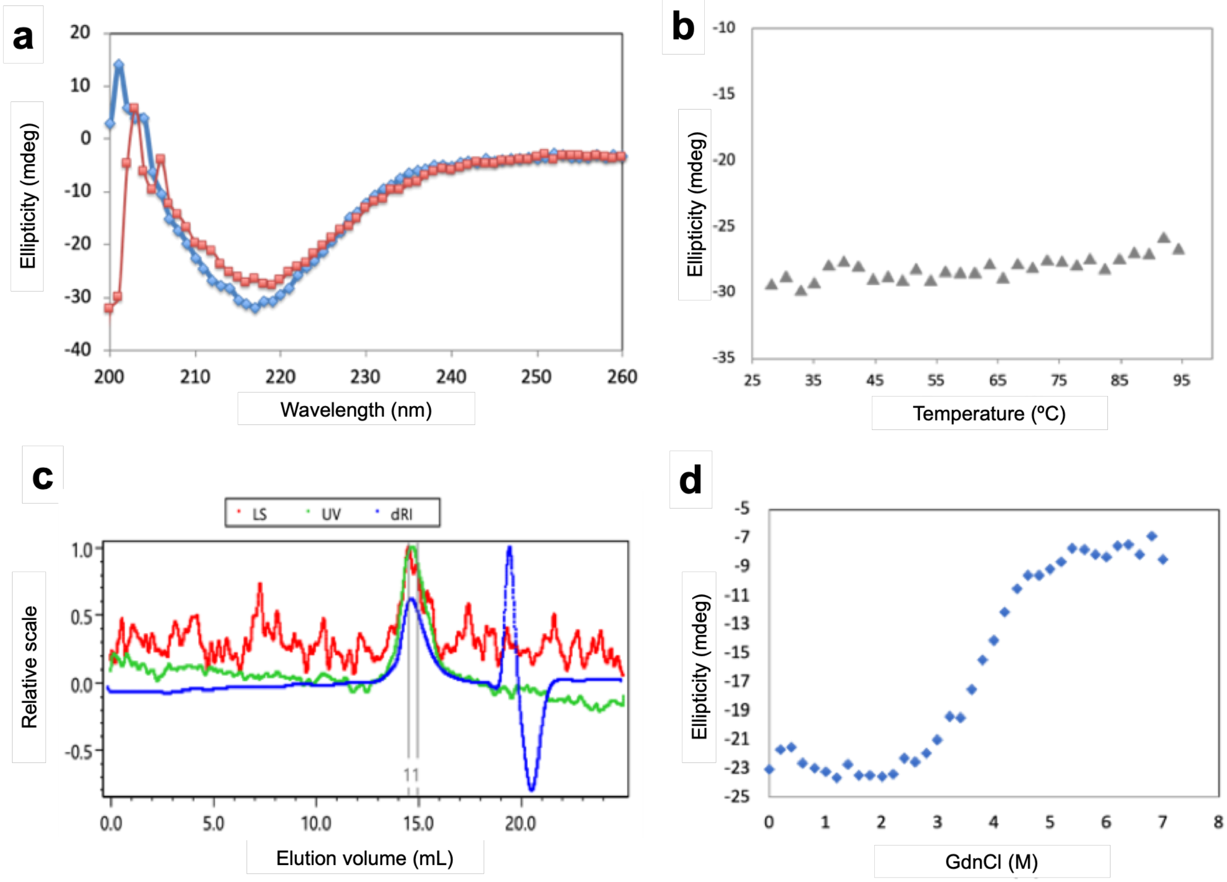

Supplementary Fig. 7. Biochemical characterization of the dIG8 design. a, Far-ultraviolet circular dichroism spectra (blue: 25 °C; red: 95 °C). **b**, Thermal denaturation monitored at 220 nm wavelength by circular dichroism. The design denatures at temperatures above 95 °C. **c**, SEC-MALS analysis showing light scattering (LS) (red), ultraviolet (UV) (green), and differential refractive index (dRI) (blue) signals. The protein is monodispersed and has an estimated molecular weight of 16.6 kDa, which lies between that corresponding to the theoretical monomer (10.3 kDa) and dimer (20.6 kDa). The protein includes the thrombin cleavage site and the hexa-histidine purification tag, which adds 2.3 kDa to the design. **d**, Chemical denaturation with guanidine hydrochloride (GdnCl) monitored at 220 nm wavelength by circular dichroism. The cooperative unfolding transition indicates that the protein is well-folded. All experiments were carried out in PBS buffer.

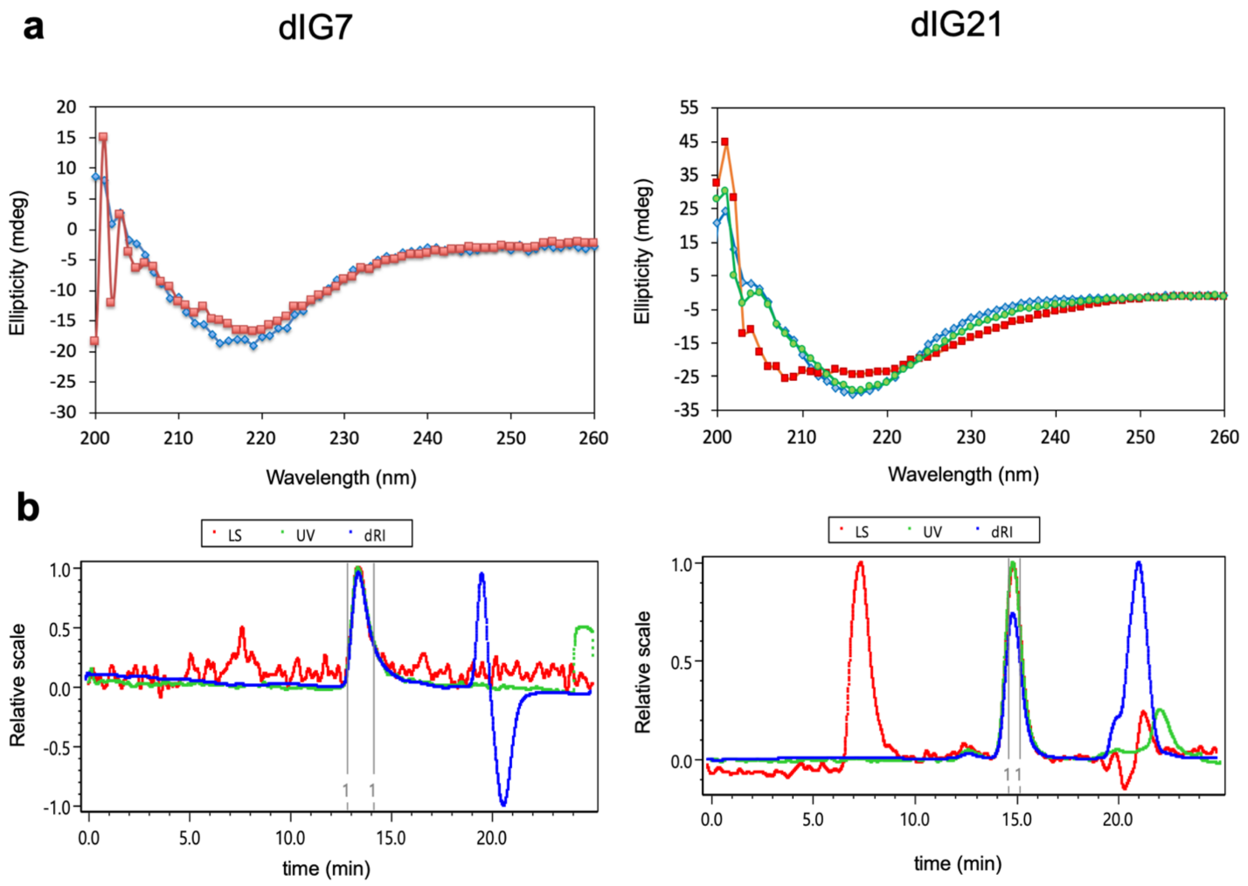

Supplementary Fig. 8. Biochemical characterization of the dIG7 and dIG21 designs. a, Far-ultraviolet circular dichroism spectra (blue: 25 °C; green, 75 °C; red: 95 °C). **b**, SEC-MALS analysis showing light scattering (LS) (red), ultraviolet (UV) (green), and differential refractive index (dRI) (blue) signals. dIG7 and dIG21 are monodispersed and have estimated molecular weights of 23.4 and 10.1 kDa, which correspond to dimer and monomer respectively. All experiments were carried out in PBS buffer.

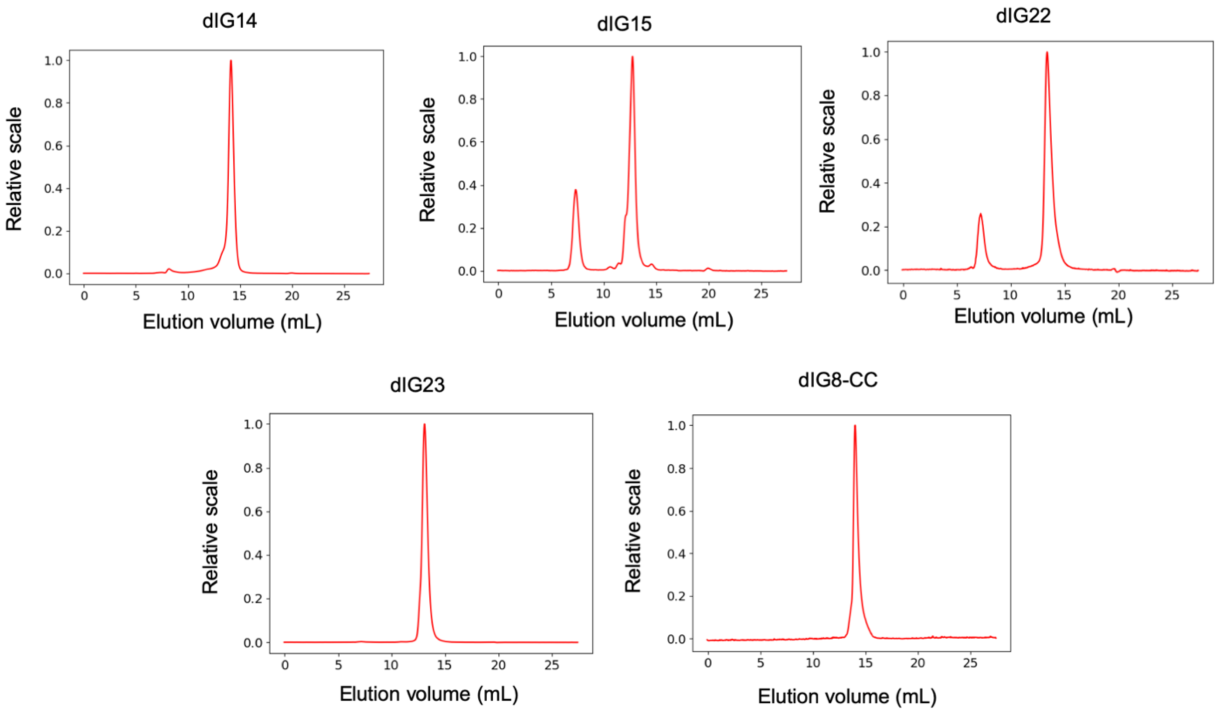

Design name	Theoretical M_w (kDa)	Estimated Mw (kDa)
dIG14	9.7	20.0±0.1
dIG15	8.8	17.5±0.3
dIG22	10.5	20.5±0.1
dIG23	10.6	20.1±0.1
dIG8-CC	8.3	12.6±0.3

Supplementary Fig. 9. Size-exclusion chromatography combined with multi-angle scattering data. dIG8-CC has a predicted molecular weight (M_w) that corresponds to between the monomer and dimer molecular weights, suggesting an equilibrium between both states. dIG14 and other representative designs are predicted to be dimers in solution. Samples were prepared in 20 mM Tris·HCl, 150 mM sodium chloride, pH 7.5.

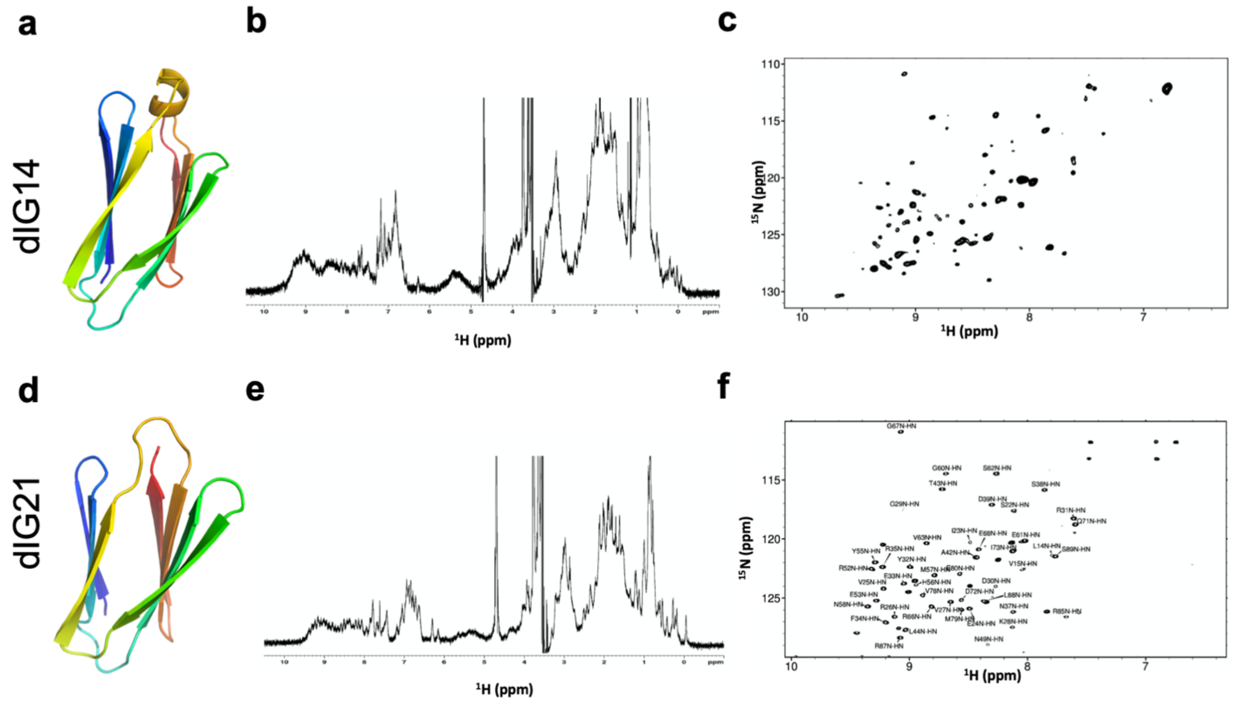

Supplementary Fig. 10. Nuclear magnetic resonance characterization of designs dIG14 and dIG21. **a**, Cartoon representation of the dIG14 design model. **b**, ^{1}H-1D spectrum showing dispersed amide (~7.5 to 9.5 ppm) and aliphatic (shifted upfield to ~ -0.2 ppm) resonances, indicative of a well-folded construct. **c**, ^{1}H-^{15}N HSQC spectrum showing considerable dispersion, consistent with the presence of significant β-extended secondary structure. **d**, Cartoon representation of the dIG21 design model. **e**, ^{1}H-1D spectrum showing dispersed amide (~ 7.5 to 9.5 ppm) and aliphatic (shifted upfield to ~ -0.1 ppm) resonances, indicative of a well-folded construct. **f**, ^{1}H-^{15}N HSQC spectrum (which are labeled where assigned) show considerable dispersion, consistent with the presence of significant β-extended secondary structure. We assigned ~ 70% of the backbone NH, C_α, C_β, CO, H_α and H_β resonances, and these assignments are in agreement with the designed secondary structure (based on predicted φ/ψ dihedral angles using TALOS+ (*2*) chemical shift analysis).

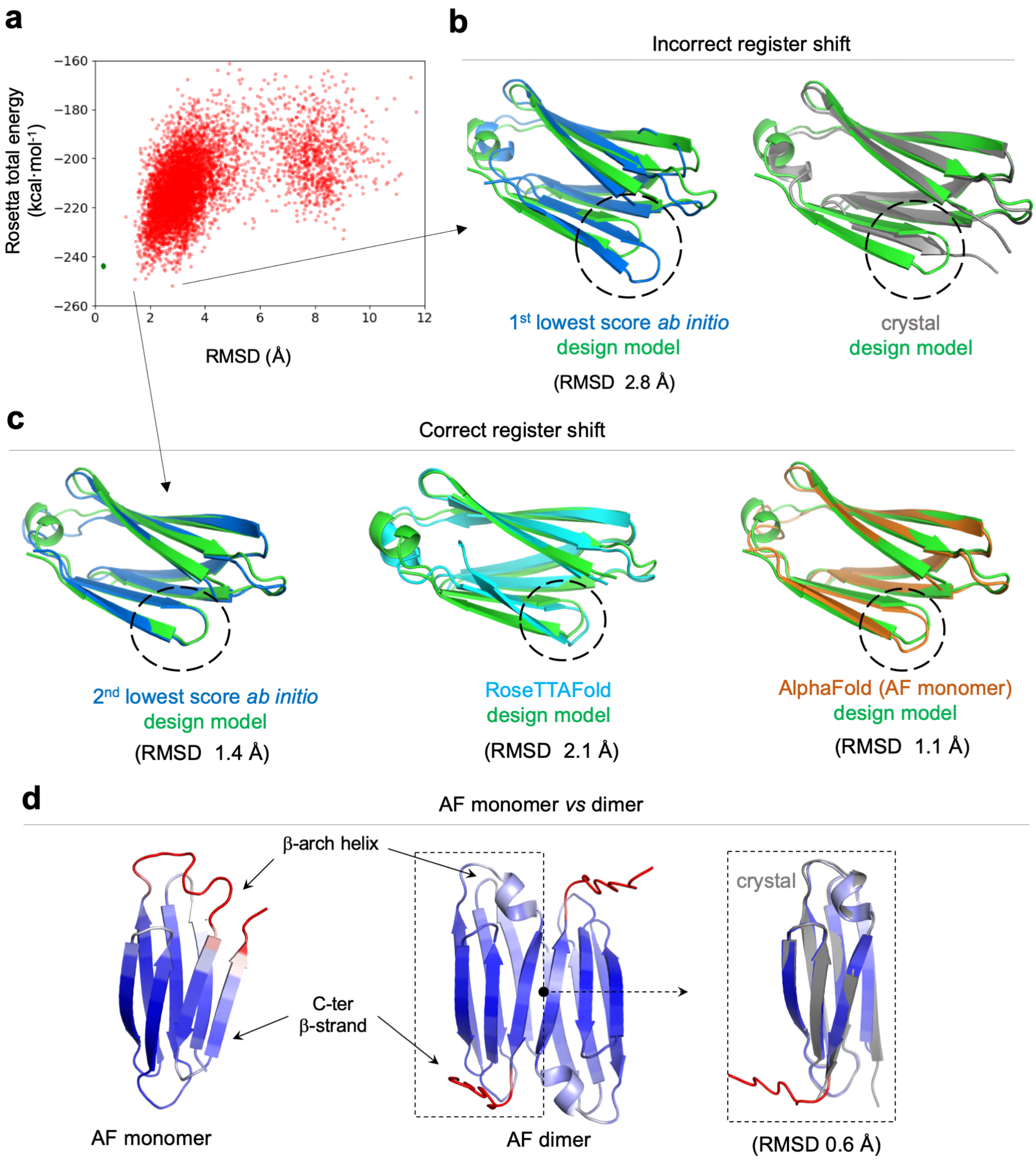

Supplementary Fig. 11. Structures predicted for design dIG14 by Rosetta ab initio folding simulations, RoseTTAFold and AlphaFold. a, b, Rosetta ab initio folding simulations (*3*) revealed that the pairing between β-strands 3 and 6 has two conformational states very close in energy, one as designed and the other as observed in the crystal structure. **c**, RoseTTAFold (*4*) and AlphaFold (*5*) predict the register shift and the C-terminal strand as designed, which disagrees with the experimental structure. None of the methods predict the C-terminal strand flip out as observed in the crystal, but all predict a conformational rearrangement of the designed β-arch helix. **d**, (left) Top AlphaFold monomer prediction colored by pLDDT (from red to blue increasing in pLDDT) highlights a sequence-structure mismatch in the β-arch

helix area. (center) Top AlphaFold dimer prediction, with monomer subunits having high pLDDT across all residues (except for the C-terminal strand residues) and matching closely the crystal structure monomer subunits (right). The predicted interface differs from the crystal structure (Fig. 4b) by a rotation of 180º between the two monomer subunits.

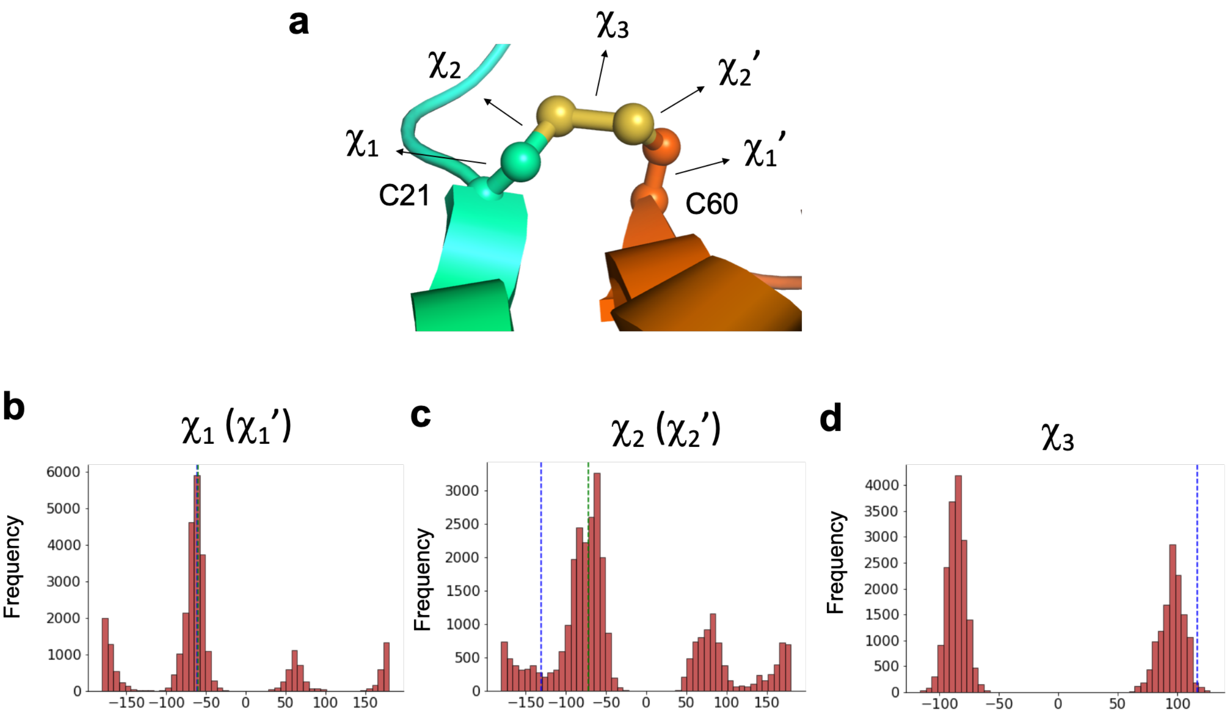

Supplementary Fig. 12. Dihedrals of the designed dIG8-CC disulfide bond in comparison with natural distributions. a, Five dihedrals describing the geometry of the designed disulfide bond (spheres and sticks) between C21 and C60. **b**, Distribution of χ_1 (and χ_1') dihedral angles obtained from a database of ~30,000 native disulfide bond geometries that was used for design (see Methods). The corresponding dihedral angles of the dIG8-CC design are represented as dashed vertical lines (χ_1 = -60.3° in blue and χ_1' = -59.9° in green). **c**, Distribution of χ_2 (and χ_2') dihedral angles obtained from the database of native disulfide bond geometries. The corresponding dihedral angles of the dIG8-CC design are represented as dashed vertical lines (χ_2 = -130.6° in blue and χ_2' = -72.0° in green). **d**, Distribution of the χ_3 dihedral angle obtained from the database of native disulfide bond geometries. The corresponding dihedral angle of the dIG8-CC design is represented as dashed vertical lines (χ_3 = 117.1° in blue). Two of the five disulfide dihedral angles (χ_2 and χ_3) are not frequently observed in distributions from naturally occurring disulfides, which is likely associated with the low disulfide bond stability suggested by the crystal structures. Source data are provided as a Source Data file.

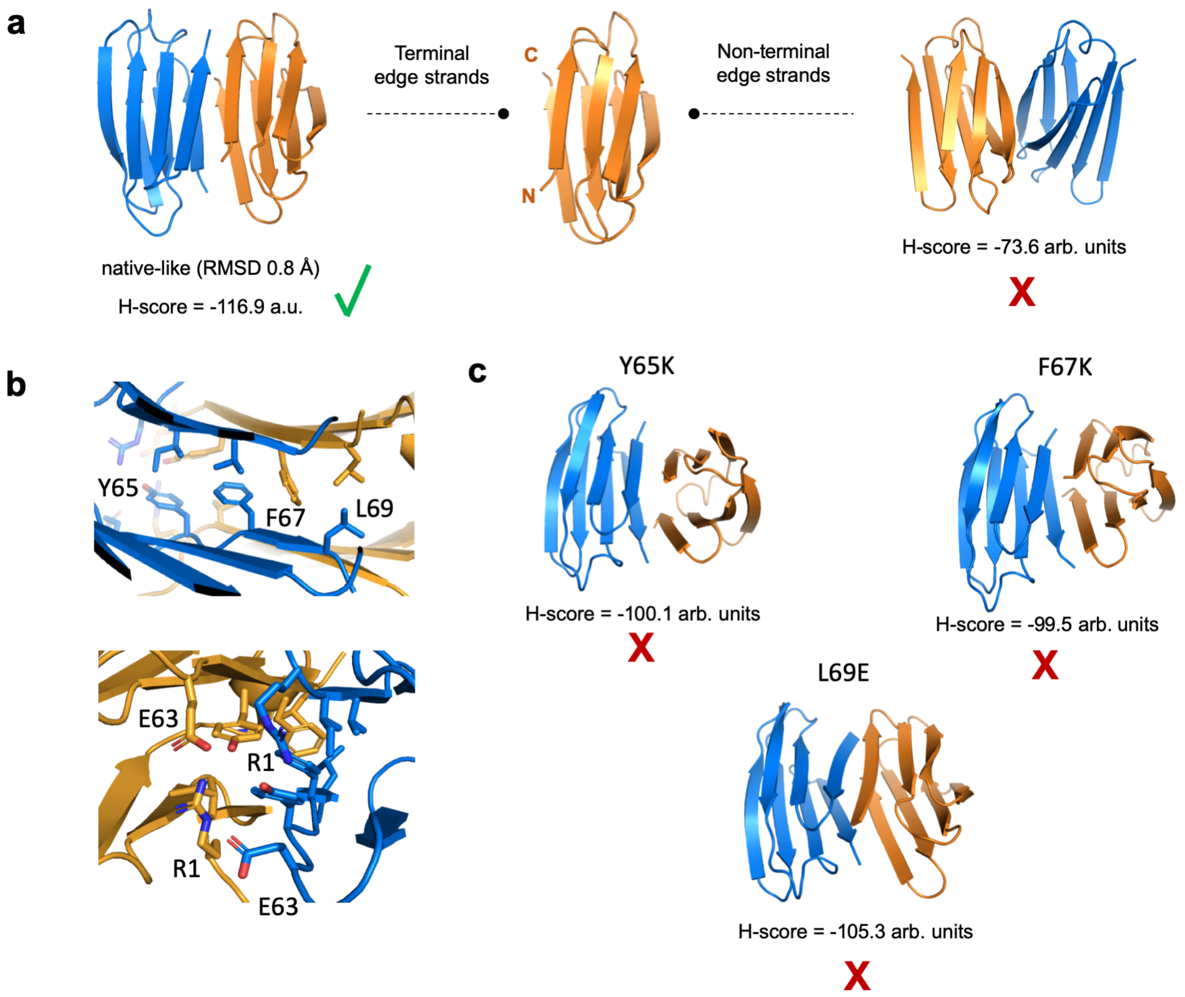

Supplementary Fig. 13. Docking calculations on the dIG8-CC homodimer interface. a, Docking calculations of two dIG8-CC monomers using ambiguous restraints between terminal edge strands recapitulate the parallel interface observed in the crystal structure (left). Docking restrained toward the opposite edge predicts dimer orientations with disrupted edge-to-edge strand pairing and worse docking scores (right); overall supporting that the terminal edge strands are more dimerization-prone. **b**, The crystal dimer interface is formed primarily by hydrophobic (top) and salt bridge (bottom) interactions. **c**, Docking calculations for single-point mutants replacing interface hydrophobics by lysine or glutamate, both of which are known to efficiently disrupt edge-to-edge interfaces as inward-pointing charged residues. All mutants are effective in disrupting the native interface. Some mutants flip the dimer orientation to form antiparallel interfaces with diminished backbone hydrogen-bonded strand pairing and overall higher docking scores. The lowest-score decoy for the most populated cluster of each simulations is shown. Docking scores (H-score), calculated with the HADDOCK docking software (*6*), are provided in arbitrary units (arb. units).

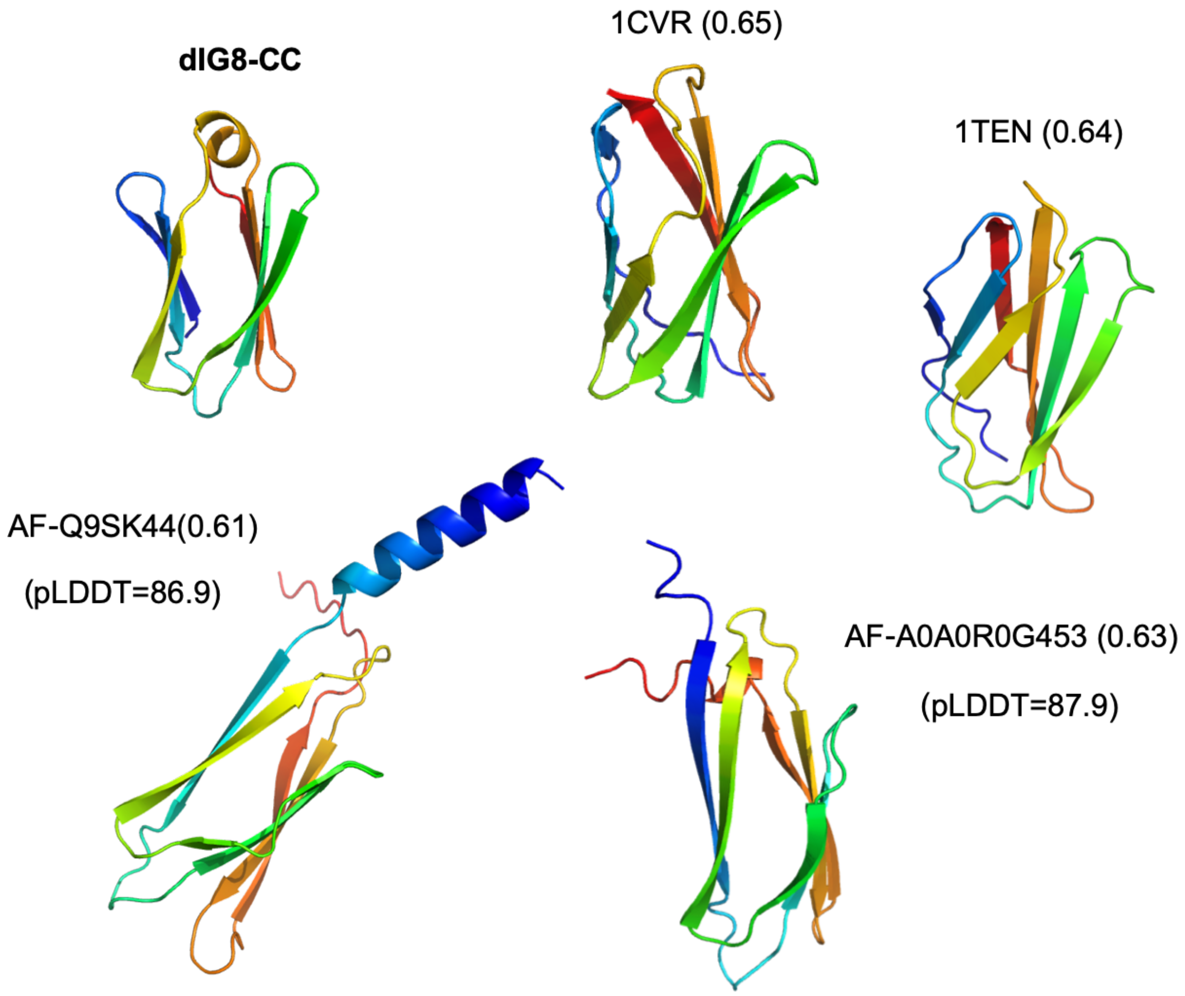

Supplementary Fig. 14. Naturally occurring protein structures most similar to design dIG8-CC found across the PDB and the AlphaFold Protein Structure Database. The closest structural analog with experimental structure available (PDB ID: 1CVR) was identified by a TM-align search over a curated dataset of immunoglobulin-like domains as identified by SCOP (*7*) (those under the Ig β-sandwich fold classification and with X-ray structure resolution < 2.5 Å). Closest structural analogues in the AlphaFold Protein Structure Database (*8*) with confident predictions (pLDDT > 85) are also shown. Normalized TM-scores are indicated in parentheses.

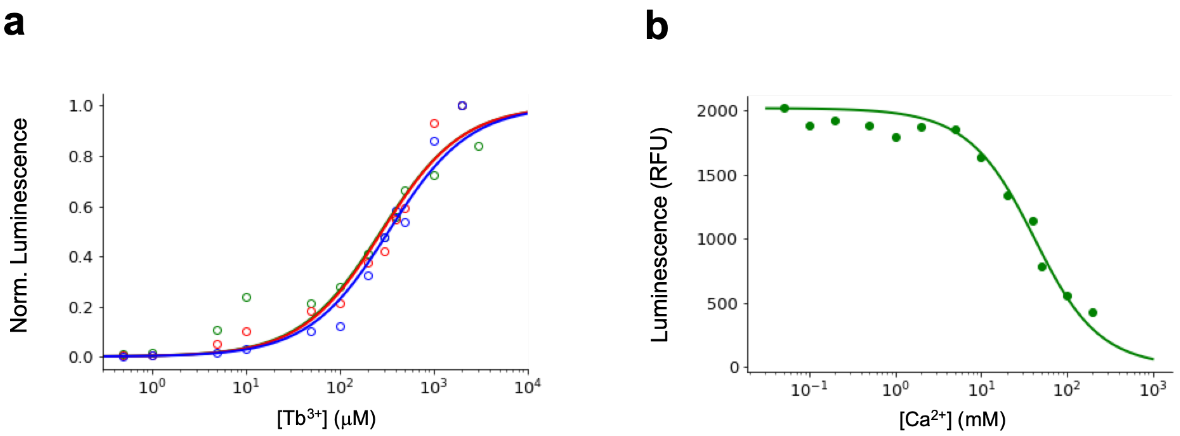

Supplementary Fig. 15. Terbium and calcium concentration-dependent luminescence of EF61_dIG8-CC. a, Normalized time-resolved luminescence intensity for Tb^{3+} titrations with EF61_dIG8-CC at three different concentrations (5 μM, green; 10 μM, red; 20 μM, blue). The three protein concentrations, each below the Tb^{3+} K_d (Fig. 5i), result in nearly identical normalized binding curves. **b**, Time-resolved luminescence intensity in relative fluorescence units (RFU) for Ca^{2+} titrations with 20 μM EF61_dIG8-CC and 100 μM Tb^{3+}, showing Ca^{2+} competition with Tb^{3+} for the EF61_dIG8-CC Tb^{3+} binding site. **a**, **b**, For each protein concentration, luminescence intensities are fit to a one-site binding model by non-linear least squares regression (lines). The excitation wavelength used was λ_{ex} = 280 nm and the emission wavelength used was λ_{em} = 544 nm. Source data are provided as a Source Data file.

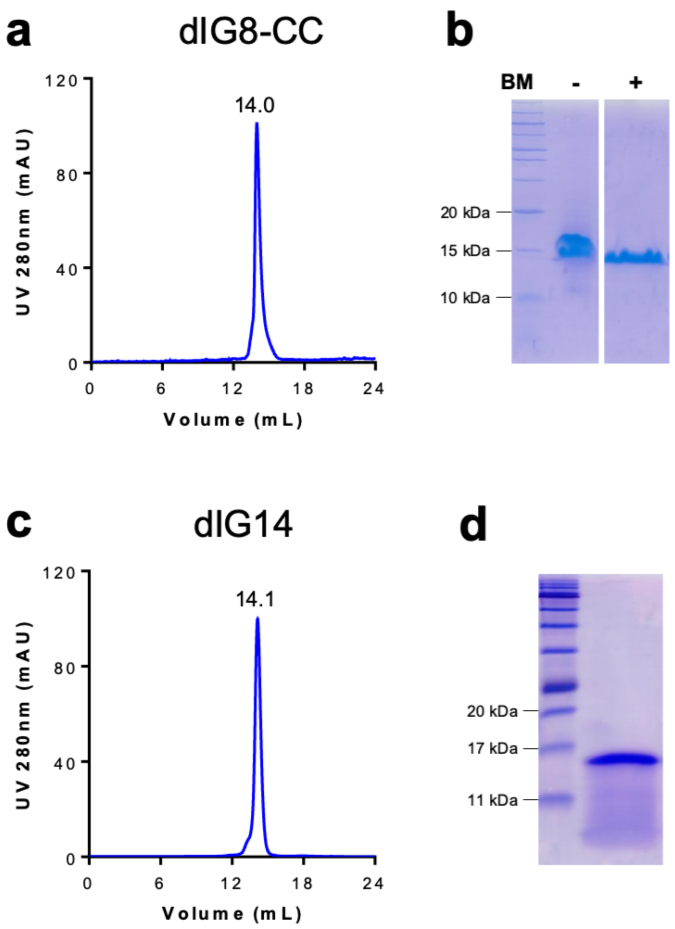

Supplementary Fig. 16. Protein purification of dIG8-CC and dIG14 for crystallization studies. Representative final size-exclusion chromatograms of dIG8-CC (**a**) and dIG14 (**c**) (n=6). Retention volumes in mL are indicated above the respective peak. Subsequent SDS-PAGE analysis of dIG8-CC (**b**) and dIG14 (**d**) after concentration (n=6), with (dIG8-CC) or without (dIG8-CC and dIG14) β-mercaptoethanol (BM).

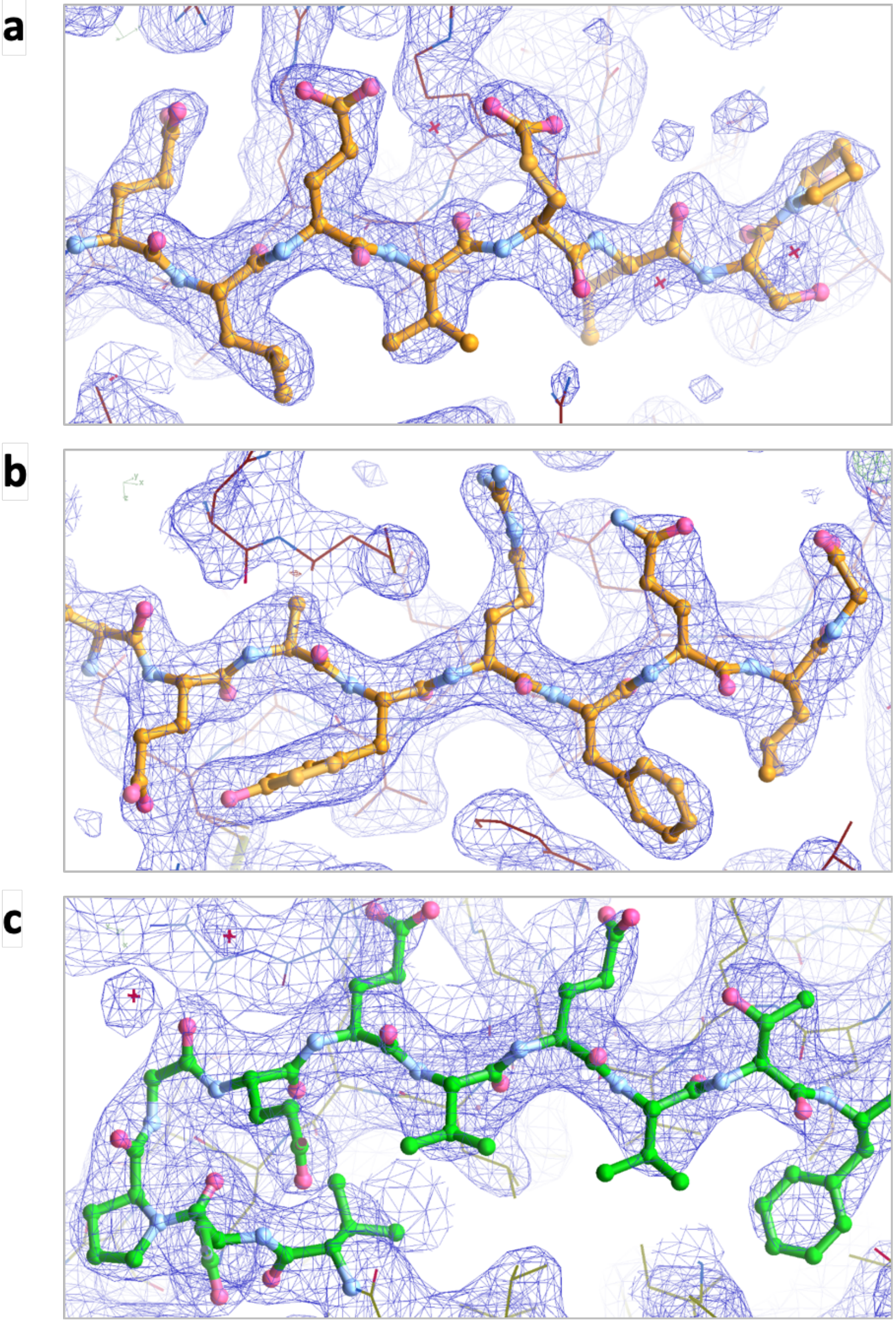

Supplementary Fig. 17. 2Fo-Fc electron density maps. (**a**) dIG8-CC (orthorhombic space group; PDB id: 7SKO), (**b**) dIG8-CC (tetragonal space group; PDB id: 7SKN), and (**c**) dIG14 (PDB id: 7SKP) are shown at a contour level of 1.0 RMSD in Coot (*7*). High-resolution cut-offs were 2.05, 2.30, and 2.50 Å, respectively, and the following sequence stretches of chain A are highlighted in ball and stick mode: (**a**) E41-P48, (**b**) N62-G70, and (**c**) V40-F50.

Supplementary Table 1. Deep-learning-based structure prediction of the designed proteins. Three metrics from the highest-confidence AlphaFold and RoseTTAFold predicted models are reported: RMSD to the design model, mean pLDDT over all residues and the minimum pLDDT value.

	AlphaFold			RoseTTAFold		
Design	RMSD (Å)	pLDDT	min(pLDDT)	RMSD (Å)	pLDDT	min(pLDDT)
dIG1	0.9	92.6	73.6	0.9	90.3	81.4
dIG2	8.9	76.3	58.7	2.2	84.1	76.3
dIG3	0.6	89.2	74.4	1.1	83.9	68.9
dIG4	0.7	92.6	69.7	0.9	86.4	61.0
dIG5	0.8	87.8	74.1	1.7	86.0	74.7
dIG6	1.1	91.0	70.5	1.3	87.8	76.0
dIG7	1.3	88.7	71.6	1.4	85.6	70.5
dIG8	1.0	90.3	77.8	0.9	86.6	66.0
dIG9	2.1	88.6	74.0	1.2	87.9	77.0
dIG10	1.2	88.6	72.3	2.3	82.0	69.5
dIG11	0.9	85.3	60.2	1.2	86.4	65.7
dIG12	3.1	84.0	52.5	1.3	88.1	77.0
dIG13	1.4	85.1	66.3	2.4	83.8	75.4
dIG14	1.2	83.6	64.8	2.0	84.0	63.1
dIG15	0.9	84.6	63.7	1.3	87.7	77.6
dIG16	1.2	90.8	73.6	2.4	78.4	55.4
dIG17	1.7	89.3	63.4	1.3	87.1	74.3
dIG18	1.6	86.0	67.7	1.8	86.2	71.0
dIG19	0.9	94.7	81.4	1.5	86.8	71.9
dIG20	2.1	83.2	61.3	1.8	79.2	49.0
dIG21	1.0	91.5	84.7	2.1	72.6	51.6
dIG22	0.8	92.6	80.8	1.2	83.5	62.8
dIG23	1.3	92.5	82.2	1.9	79.4	58.9
dIG24	0.7	88.2	76.4	2.0	86.3	69.4
dIG25	1.3	85.2	67.6	2.3	85.0	69.8

dIG26	0.9	85.7	65.7	2.1	83.8	58.1
dIG27	1.2	85.2	52.7	1.5	86.5	76.4
dIG28	1.1	89.3	72.4	2.0	87.4	69.4
dIG29	0.7	92.7	73.7	2.3	78.0	68.3
dIG30	1.2	84.1	67.8	1.9	85.4	74.0
dIG31	1.0	84.7	69.5	1.4	86.2	71.2

Supplementary Table 2. Designed protein sequences in comparison with naturally occurring ones. The lowest E-values obtained from BLAST (*9*) (against the NCBI nr database of non-redundant protein sequences), and more sensitive sequence-profile searches with HHBlits (*10*) (against the UniRef30 database) and HHPred (*11*) are reported. The PDB ID of the lowest E-value hit identified with HHPred is also shown in parentheses.

Design	Amino acid sequence	Blast	HHBlits	HHpred
dIG1	TVEVRIRKNGNEYEVEVENRSDRPAEVRF HYDGTTETYTVPPGTRLRYRTKLTKPMRIE VRAGNTTYEYTVS	0.1	0.073	0.057 (2r39)
dIG2	EIHVELRKEGDRVEVRVENRSSQPGTVEIE VDGQRYEFTANPGERIQFEARGKTPVRVE VVYGNTTYRYEVR	3.8	0.19	0.056 (2r39)
dIG3	RVRVEVKNNKIEVENNSDQPAEIHLEFGGR RFTYTGNKGERIEVQISPEEAKNARIEIKVG DKKLEYQYH	3.5	2.9	4.1 (4ktp)
dIG4	RVEVRISGNTIRVENRSDRPARVEFEYGGR REEYTAPPGSELRVTISPEELKNARVEIEYG GQRYRFEVT	0.73	1.6	6.7 (1r0u)
dIG5	KIRIEVRSSGNTIHVEVENNSDRPVRIRVTA PGTTLETTANPGERVRFEFRGVPPGGEVEV EVKAGDEKVRTRYRS	0.7	0.067	0.54 (2x3c)
dIG6	TVEVRITEKNGQWEVRIRNRSSQPARVEVE EGGRREEYTLNPGDELELHFTSPKPVRITV EVGGQRYTYTLR	1.2	0.5	0.92 (4xin)
dIG7	RMEVRVSNGRVEIENKSSQPGRVEVRFNG KRYEYTANPGERVEVEVSPEELKNLRVRL EYDGKTEETQYS	2.1	0.056	0.47 (6w0p)
dIG8	RIEVRVDNGRVRVRNGTDRPVRVRVTAGG ETREYTVNPGTELEVELSPEQQNNAEVEVE VGNEKYRFQLG	3.8	0.47	3.0 (6w0p)
dIG9	SIRVEIEKRGDSYRVEVENRSDQPAEIEVR WNGRRERYEANKGETVEVEVRAPSPVEVR VRAGNTEVRVEQR	1.0	0.47	0.46 (2r39)
dIG10	RVEVRISGNTIEIRSEGPGRLELEYNGQREE YTLNPGTRIEFEGRPGEEVRVEVEMNGQR YTFEVRF	1.6	0.32	0.44 (6w0p)
dIG11	RLEVRMEGKKVEVRNNSDRPMRVEFTWN GQRERYHVNPGETLEVEVQPGARVEVRVQ SGDTQYRYEFEL	2.1	0.061	0.089 (6e5c)
dIG12	SLEVRVRKSGNTFEVEIRNKSDRPAEVRLEI GGRRETYTVPPGSTLRLRGPGKRPGRVEIK AGDAKYEVELR	0.62	0.11	0.012 (2r39)

dIG13	YVEIRYKGEKVHIRTNGPVTLEVEFEGKRE RYTLNPGEELEIRIRARRIRVEVQEGDRKIE TELTF	0.31	0.2	0.41 (6e5c)
dIG14	RVEVRVEFEGDKMRVRLRNDSSTPVEVHI KVGDEKRTVTVNPGEEVEVTFSANDPHKF NRPQFTIEWGGQRQHFQHH	0.72	0.0028	0.26 (4ay0)
dIG15	RPKVQLELHGNKMRVRLRNDSSTPVEVHI KVGDEKRTVTVNPGEEVEVTFSTTDPREL KNATIQLHQGDQTVEYRVD	0.2	0.0031	0.63 (2r39)
dIG16	EVEIEVRTKNGKIEVRVTNRSDRPVEVRME KGGQRETYTAPPGSTVRVEFSPSDDRQKRP TVEVTVNGRRYEVRVH	2.5	0.22	0.044 (5ngl)
dIG17	RVEFRLREEGDRYRLEIRTDRPGTIEIEVNG RRERYTANPGTTITVEGTRGEEVEVTVEYD GKRERWRFRM	1.0	0.72	7.7 (6ex6)
dIG18	RVRWTWRISGNTIEFRFENNSDRPARVEIE VDGQRREYTVNPGERLELHFQAGAREIRV EVEVGKEKYEVRIRF	0.51	0.056	0.37 (2r39)
dIG19	RVEVRIREEGDKYELRIRNRSDRPAEVRIE KGGKRETYTVNPGEELRIEFPPGAPPGRVE VQVGDKKYEYTVK	1.8	0.065	0.39 (6i60)
dIG20	VVEVRLEGERIRVRNNSDRPATVHVEKDG QRETYTVNPGEELEITSPDSSQNKGLRLRIH VEVNGQRFTFEFTM	0.51	0.024	3.6 (6w8u)
dIG21	SIEVRVKGDRYEFRNNSDKPATLEVEKNG KREEYHMNPGESVEVRGEPGQDIRFEMVM EGTTYRYRLS	0.61	0.044	1.7 (7agw)
dIG22	SIEVRVKGDRYEFRNNSDKPATLEVEKNG KREEYHMNPGESVEVRGPPGQDIRFEMTM DGTTYRYRLS	3.6	0.31	1.7 (7agw)
dIG23	DLEVRRKDGKFEFRNNSDKPATLEVEKDG QREEYRMNPGETIEVQAPPGQDVRFTVEM PGREYRYKLD	1.0	0.021	0.16 (3q48)
dIG24	TFEVRVQWSGNTIRVTVENQSDRPATVRIE YGNTTYQRTINPGDRLTVEFTGGPGEVHV EVEINGKREERTFTK	4.5	0.032	0.35 (3sd2)
dIG25	EVQMRVEISGDTIRVEVRNNSDRPGRVEFE VGGVRTSYTMNPGERIEVEVTVSTAEKQGI KVEVHVEAGDEKRTYEFQM	2.3	0.99	0.83 (6fjy)

dIG26	RVEVRVQEKNGKVEIRVRSDGPVRVEVEVGGQRREYTGNPGEEVEIEVTADQPVRVEVKAGDKKFTYTVSE	2.1	0.36	15 (6w0p)
dIG27	MFRVEVREKNGRVEVRVENRSDRPGTVEVEVGGVRLRFTVNPGEELEIRMDVPNGRRVEIEIVGKGVKYSYEYTV	1.3	0.13	1.6 (2wnw)
dIG28	SWEVRVRWKNGRLEVEIRNNSSQPGKVRIEFDGKRHEVHLNPGESTKWRFENPGGEFHVEAGKEKYTYTV	2.5	0.017	1.3 (2wnw)
dIG29	RVEVRQSGNTIEIRSEGPGRLELEYNGQREEYTLNPGTRYEYEGRPGEEVRVEVEMNGQRYTYEVRS	2.8	1.0	1.5 (5bvq)
dIG30	RSEVHVRFEGERIEIQIHNGTDKPARVEMEVNGQRYEYHMPPNSKMEYRVPLRQEIRFEVEVGGQRFTYRYTS	2.8	0.65	2.3 (1v7w)
dIG31	RVEVRVTYKGNRVEVRVRNNSDRPVRFRVVGPGAKYELKGNPGTEMRVEIRVPNAREIEVEVNGQRQRYQM	4.1	0.94	1.8 (6ywf)
dIG8-CC	RIEVRVDNGRVRVRNGTDRPCRVRVTAGGETREYTVNPGTELEVELSPEQQNNAEVEVECGNEKYRFQLG	3.3	0.033	2.1 (6w0p)
EF61_dIG8-CC	RIEVRVDNGRVRVRNGTDRPCRVRVTAGGETREYTVNPGTELEVELSPEQQNNAEVEVECTVDDKDGDGYISAAEAAVEKYRFQLG	8.7	0.006	0.0018 (6ohh)

Supplementary Table 3. Cross-β geometrical parameters calculated for the designed proteins. For comparison, median and median absolute deviation values for cross-β parameters calculated from naturally occurring Ig domain structures are also provided, as shown in Supplementary Fig. 3a.

Design	Distance (Å)	Twist (°)	Roll (°)	Tilt (°)
dIG1	11.5	-3.4	12.6	-6.4
dIG2	10.7	-9.2	14.6	6.8
dIG3	11.3	-12.4	20.5	27.1
dIG4	11.0	-10.8	10.6	7.2
dIG5	10.2	-24.7	-5.7	-8.3
dIG6	11.2	5.5	4.3	-4.8
dIG7	10.4	-18.7	-7.4	-18.8
dIG8	10.0	-16.6	6.8	3.1
dIG9	10.3	-13.9	11.6	8.3
dIG10	11.0	-8.6	6.6	-2.7
dIG11	11.1	-17.1	5.2	8.1
dIG12	10.7	2.0	11.0	4.0
dIG13	11.5	-19.2	-4.7	-10.4
dIG14	11.2	-13.8	9.9	4.4
dIG15	11.0	-16.8	11.3	-5.1
dIG16	10.5	-14.5	-4.1	-12.5
dIG17	9.8	-2.6	-4.9	-0.3
dIG18	12.2	-1.8	-0.1	-2.0
dIG19	11.2	-5.7	19.2	1.8
dIG20	10.8	11.9	-9.8	-2.9
dIG21	10.9	-18.4	-17.6	-19.0
dIG22	10.7	4.5	9.9	-5.8
dIG23	10.8	0.3	3.2	-1.1
dIG24	10.3	-20.7	-8.8	-12.5
dIG25	10.0	-21.5	-0.7	-17.4
dIG26	12.2	0.6	20.0	16.1

dIG27	10.8	-15.7	6.1	10.7
dIG28	10.4	1.2	5.5	1.3
dIG29	11.0	-8.6	6.7	2.7
dIG30	10.8	-18.2	6.5	-7.1
dIG31	11.9	-7.9	13.5	26.5
**Natural Ig domains**	**10.9 ± 0.8**	**-32.1 ± 7.7**	**12.0 ± 12.2**	**4.0 ± 11.1**

Supplementary Table 4. Summary of the experimental characterization of designs.

dIG	Soluble expression	Monodisperse	CD* spectra (25 °C)	T_m‡ (°C)	Oligomeric state†
1	No	-	-	-	-
2-6,9,11-13, 16-19,24-31	Yes	No	-	-	-
10,20	Yes	Yes	β	> 95°C	High
7,14,15,22,23	Yes	Yes	β	> 95°C	D
21	Yes	Yes	β	> 75°C	M
8,8-CC	Yes	Yes	β	> 95°C	M/D

* 'CD', circular dichroism. ‡ 'T_m', melting temperature. † Oligomeric state of the dominant species determined with size-exclusion chromatography with multi-angle light-scattering (SEC-MALS) ('M', monomer; 'D', dimer).

Supplementary Table 5. Crystallographic data.

Dataset	dIG8-CC (tetragonal)	dIG8-CC (orthorhombic)	dIG14
Beam line (synchrotron)	ID30A-3 (ESRF)	XALOC (ALBA)	XALOC (ALBA)
Space group / complexes per a.u. [a]	$P4_12_12$ / 4	$C222_1$ / 4	$P4_32_12$ / 2
Cell constants (a,b and c in Å)	58.17, 58.17, 173.50	43.03, 76.52, 165.80	73.91, 73.91, 97.48
Wavelength (Å)	0.96770	0.97879	0.97918
Measurements / unique reflections	409,652 / 13,994	221,806 / 17,590	481,439 / 9805
Resolution range (Å) (outermost shell) [c]	55.2 – 2.30 (2.43 – 2.30)	38.3 – 2.05 (2.17 – 2.05)	58.9 – 2.50 (2.65 – 2.50)
Completeness (%) / R_{merge} [d]	99.9 (99.7) / 0.178 (2.829)	99.6 (98.0) / 0.101 (2.453)	99.6 (99.3) / 0.096 (3.057)
R_{pim} [e] / CC($^1/_2$) [e]	0.033 (0.520) / 0.999 (0.865)	0.030 (0.781) / 1.000 (0.601)	0.014 (0.435) / 0.999 (0.901)
Average intensity [f]	15.2 (1.5)	15.2 (1.3)	30.2 (3.2)
B-Factor (Wilson) (Å^2) / Aver. multiplicity	58.4 / 29.3 (30.6)	56.2 / 12.6 (10.9)	86.2 / 49.1 (50.4)
Resolution range used for refinement (Å)	37.2 – 2.30	38.3 – 2.05	58.9 – 2.50
Reflections used (test set)	13,415 (511)	16,855 (706)	9429 (376)
Crystallographic R_{factor} (free R_{factor}) [d]	0.263 (0.301)	0.250 (0.291)	0.247 (0.300)
Non-H protein atoms/waters/ligands per a.u.	2316 / 22 / -	2138 / 34 / 1 Mg^{2+}	1190 / 15 / -
Rmsd from target values			
bonds (Å) / angles (°)	0.002 / 0.48	0.002 / 0.43	0.008 / 0.87
Average B-factor (Å^2)	63.7	63.2	94.6
Protein contacts and geometry analysis [b]			
Ramachandran favored / outliers / all analysed	284 (98.3%) / 1 / 289	279 (100%) / 0 / 279	142 (100%) / 0 / 142
Bond-length / bond-angle / chirality / planarity outliers	0 / 0 / 0 / 0	0 / 0 / 0 / 0	0 / 0 / 0 / 0
Side-chain outliers	8 (3.1%)	2 (0.9%)	8 (6.0%)
All-atom clashes / clashscore [b]	18 / 3.9	16 / 3.8	11 / 4.7
RSRZ outliers / F_o:F_c correlation	16 (5.5%) [b] / 0.93	15 (5.3%) [b] / 0.95	12 (8.5%) [g] / 0.93
PDB access code	7SKN	7SKO	7SKP

[a] Abbreviations: a.u., asymmetric unit; PEG, diethylene glycol; RSRZ, real-space R-value Z-score. [b] According to the wwPDB Validation Service (https://wwpdb-validation.wwpdb.org/validservice). [c] Values in parenthesis refer to the outermost resolution shell if not otherwise indicated. [d] For definitions, see Table 1 in (*12*). [e] For definitions, see (*13*, *14*). [f] Average intensity is <I/σ(I)> of unique reflections after merging according to Xscale (*15*). [g] According to Coot (<0.15; (*7*)).

Supplementary Table 6. Occupancies for the bound and unbound disulfides between C21 and C60 observed in the crystal structures of dIG8-CC. Neither rotamer nor Ramachandran outliers were identified for the cysteines across the eight protomers. Despite possible radiation damage and partial reduction from the hexahistidine tag removal step with TEV-protease (see Methods), the close to 50% formation suggests low disulfide bond stability.

Crystal structure	Chain	Disulfide-bonded	Unbound
$P4_12_12$ (7SKN)	A	0.45	0.55
	B	0.50	0.50
	C	0.55	0.45
	D	0.38	0.62
$C222_1$ (7SKP)	A	0.48	0.52
	B	0.50	0.50
	C	0.00	1.00
	D	0.67	0.33

Supplementary Table 7. Primers for the second generation of plasmids

pET28*-dIG8-CC	
Forward	CATGGGCAGCAGCCATCATCATCATCATCACAGCAGCGGCGAAAACCTTTATTTTCAGGGCCA
Reverse	TATGGCCCTGAAAATAAAGGTTTTCGCCGCTGCTGTGATGATGATGATGATGGCTGCTGCC
pET28*-dIG14	
Forward	ATGCCCATGGGCCGTGTTGAGGTGC
Reverse	GCATCTCGAGTTAATGGTGATGGTGGTGGTGTTGAAAGTG

References

1. Y. Zhang, Skolnick, J., TM-align: a protein structure alignment algorithm based on the TM-score. Nucleic Acids Res. 33, 2302–2309 (2005).

2. Y. Shen, F. Delaglio, G. Cornilescu, A. Bax, TALOS+: a hybrid method for predicting protein backbone torsion angles from NMR chemical shifts. J. Biomol. NMR. 44, 213–223 (2009).

3. P. Bradley, K. M. S. Misura, D. Baker, Toward High-Resolution de Novo Structure Prediction for Small Proteins. Science. 309, 1868–1871 (2005).

4. M. Baek, F. DiMaio, I. Anishchenko, J. Dauparas, S. Ovchinnikov, G. R. Lee, J. Wang, Q. Cong, L. N. Kinch, R. D. Schaeffer, C. Millán, H. Park, C. Adams, C. R. Glassman, A. DeGiovanni, J. H. Pereira, A. V. Rodrigues, A. A. van Dijk, A. C. Ebrecht, D. J. Opperman, T. Sagmeister, C. Buhlheller, T. Pavkov-Keller, M. K. Rathinaswamy, U. Dalwadi, C. K. Yip, J. E. Burke, K. C. Garcia, N. V. Grishin, P. D. Adams, R. J. Read, D. Baker, Accurate prediction of protein structures and interactions using a three-track neural network. Science. 373, 871–876 (2021).

5. J. Jumper, R. Evans, A. Pritzel, T. Green, M. Figurnov, O. Ronneberger, K. Tunyasuvunakool, R. Bates, A. Žídek, A. Potapenko, A. Bridgland, C. Meyer, S. A. A. Kohl, A. J. Ballard, A. Cowie, B. Romera-Paredes, S. Nikolov, R. Jain, J. Adler, T. Back, S. Petersen, D. Reiman, E. Clancy, M. Zielinski, M. Steinegger, M. Pacholska, T. Berghammer, S. Bodenstein, D. Silver, O. Vinyals, A. W. Senior, K. Kavukcuoglu, P. Kohli, D. Hassabis, Highly accurate protein structure prediction with AlphaFold. Nature. 596, 583–589 (2021).

6. G. C. P. van Zundert, J. P. G. L. M. Rodrigues, M. Trellet, C. Schmitz, P. L. Kastritis, E. Karaca, A. S. J. Melquiond, M. van Dijk, S. J. de Vries, A. M. J. J. Bonvin, The HADDOCK2.2 Web Server: User-Friendly Integrative Modeling of Biomolecular Complexes. J. Mol. Biol. 428, 720–725 (2016).

7. A. Casañal, B. Lohkamp, P. Emsley, Current developments in Coot for macromolecular model building of Electron Cryo-microscopy and Crystallographic Data. Protein Sci. 29, 1055–1064 (2020).

8. K. Tunyasuvunakool, J. Adler, Z. Wu, T. Green, M. Zielinski, A. Žídek, A. Bridgland, A. Cowie, C. Meyer, A. Laydon, S. Velankar, G. J. Kleywegt, A. Bateman, R. Evans, A. Pritzel, M. Figurnov, O. Ronneberger, R. Bates, S. A. A. Kohl, A. Potapenko, A. J. Ballard, B. Romera-Paredes, S. Nikolov, R. Jain, E. Clancy, D. Reiman, S. Petersen, A. W. Senior,

K. Kavukcuoglu, E. Birney, P. Kohli, J. Jumper, D. Hassabis, Highly accurate protein structure prediction for the human proteome. Nature. 596, 590–596 (2021).

9. C. Camacho, G. Coulouris, V. Avagyan, N. Ma, J. Papadopoulos, K. Bealer, T. L. Madden, BLAST+: architecture and applications. BMC Bioinformatics. 10, 421 (2009).

10. M. Remmert, A. Biegert, A. Hauser, J. Söding, HHblits: lightning-fast iterative protein sequence searching by HMM-HMM alignment. Nat. Methods. 9, 173–175 (2012).

11. L. Zimmermann, A. Stephens, S.-Z. Nam, D. Rau, J. Kübler, M. Lozajic, F. Gabler, J. Söding, A. N. Lupas, V. Alva, A Completely Reimplemented MPI Bioinformatics Toolkit with a New HHpred Server at its Core. J. Mol. Biol. 430, 2237–2243 (2018).

12. R. García-Castellanos, A. Marrero, G. Mallorquí-Fernández, J. Potempa, M. Coll, F. X. Gomis-Rüth, Three-dimensional Structure of MecI: Molecular basis for transcriptional regulation of staphylococcal methicillin resistance. J. Biol. Chem. 278, 39897–39905 (2003).

13. M. S. Weiss, Global indicators of X-ray data quality. J. Appl. Crystallogr. 34, 130–135 (2001).

14. P. A. Karplus, K. Diederichs, Linking Crystallographic Model and Data Quality. Science. 336, 1030–1033 (2012).

15. W. Kabsch, XDS. Acta Crystallogr. D Biol. Crystallogr. 66, 125–132 (2010).
