# Supplementary material for: De novo design of immunoglobulin-like domains
Source: Nat Commun. 2022 Oct 3;13:5661. doi: 10.1038/s41467-022-33004-6 (PMC9530121; doi:10.1038/s41467-022-33004-6)
Supplement: Supplementary file 3 — Description of Additional Supplementary Files [file 41467_2022_33004_MOESM3_ESM.pdf]

Description of Additional supplementary files

File name: Supplementary Data 1

Description: Experimentally tested designed protein structures.
